# Spatiotemporal absorption fluctuation imaging based on U-Net

**DOI:** 10.1117/1.JBO.27.2.026002

**Published:** 2022-02-08

**Authors:** Min Yi, Lin-Chang Wu, Qian-Yi Du, Cai-Zhong Guan, Ming-Di Liu, Xiao-Song Li, Hong-Lian Xiong, Hai-Shu Tan, Xue-Hua Wang, Jun-Ping Zhong, Ding-An Han, Ming-Yi Wang, Ya-Guang Zeng

**Affiliations:** a Guangdong-Hong Kong-Macao Joint Laboratory for Intelligent Micro-Nano Optoelectronic Technology, Foshan, China; b Foshan University, School of Physics and Optoelectronic Engineering, Foshan, China; c Guangdong Provincial Key Laboratory of Animal Molecular Design and Precise Breeding, Foshan, China

**Keywords:** optical angiography, spatiotemporal absorption fluctuation imaging, dynamic blood flow imaging, U-Net

## Abstract

**Significance:**

Full-field optical angiography is critical for vascular disease research and clinical diagnosis. Existing methods struggle to improve the temporal and spatial resolutions simultaneously.

**Aim:**

Spatiotemporal absorption fluctuation imaging (ST-AFI) is proposed to achieve dynamic blood flow imaging with high spatial and temporal resolutions.

**Approach:**

ST-AFI is a dynamic optical angiography based on a low-coherence imaging system and U-Net. The system was used to acquire a series of dynamic red blood cell (RBC) signals and static background tissue signals, and U-Net is used to predict optical absorption properties and spatiotemporal fluctuation information. U-Net was generally used in two-dimensional blood flow segmentation as an image processing algorithm for biomedical imaging. In the proposed approach, the network simultaneously analyzes the spatial absorption coefficient differences and the temporal dynamic absorption fluctuation.

**Results:**

The spatial resolution of ST-AFI is up to 4.33  μm, and the temporal resolution is up to 0.032 s. In vivo experiments on 2.5-day-old chicken embryos were conducted. The results demonstrate that intermittent RBCs flow in capillaries can be resolved, and the blood vessels without blood flow can be suppressed.

**Conclusions:**

Using ST-AFI to achieve convolutional neural network (CNN)-based dynamic angiography is a novel approach that may be useful for several clinical applications. Owing to their strong feature extraction ability, CNNs exhibit the potential to be expanded to other blood flow imaging methods for the prediction of the spatiotemporal optical properties with improved temporal and spatial resolutions.

## 1 Introduction

Several attempts have been made to integrate optical techniques with existing blood flow imaging methods, with varying degrees of success. Techniques based on point-by-point scanning modes, such as two-photon imaging and optical coherence angiography,^1^^,^^2^ have been applied to obtain high-quality optical angiography images. However, signals acquired at different scanning positions are not synchronous, and scanning mode cannot provide full-field information of blood microcirculation at the same time.^3^ To address this issue, several full-field optical imaging methods have been developed for blood flow imaging. For example, Hong et al. performed real-time, full-field epifluorescence imaging of mouse hindlimb vasculatures in the second near-infrared region (i.e., 1000 ∼ 1700 nm)^4^; however, the variability in factors, such as fluorophore concentration, optical quality of the sample, system calibration, and photobleaching, affects the imaging performance.^5^ More recently, Hong et al. presented a new model for temporal speckle contrast imaging; this model includes derivations for the expectation and fluctuation in the temporal speckle contrast, thereby providing a guideline for selecting suitable statistical sample sizes for the application of the temporal speckle contrast imaging.^6^ Furthermore, an optical method that combines laser speckle contrast imaging (LSCI) with microendoscopy to enable time-lapse blood flow detection in deep regions of the brain has been developed by Chen et al.^7^ Moreover, Sang et al. combined the idea of separating dynamic from static light scattering using optical transparency technology, to develop a dynamic speckle imaging method that improves sample imaging depth.^8^ However, LSCI is affected by not only the concentration and velocity of moving particles, but also low-frequency background noise.^9^ In our previous work, we developed a full-field optical angiography method using principal component analysis (PCA). This method can effectively extract blood flow signals from a limited-frame raw image and considerably improve the temporal resolution.^10^ Unfortunately, this method is not sufficiently sensitive to detect small blood vessels and requires several raw images to analyze the components of microvessels. Therefore, the development of a high-spatial-resolution blood flow imaging method that can function effectively for a limited number of raw blood flow images is imperative.

In recent years, deep learning has achieved significant success in the field of biological imaging. Convolutional neural networks (CNNs) have been used for image processing to analyze the results of a few optical imaging methods, such as optical coherence tomography, photoacoustic imaging, and fluorescence imaging, to realize vessel segmentation and classification,^11^^–^^16^ cell morphometry,^17^^,^^18^ and ocular disease analysis and diagnosis.^19^^–^^25^ Further, CNNs have been used in combination with abiotic optical imaging methods to reconstruct image information by analyzing optical properties. Zhu et al. introduced a physics-informed learning method for imaging through unknown diffusers by combining physics-based theories with CNNs.^26^ Zheng et al. proposed an end-to-end deep neural network to detect and identify unique features in incoherent images. This method enables single-shot incoherent imaging in highly nonstatic and optically thick turbid media.^27^ Tong et al. established an accurate propagation model of the optical imaging system using a deep CNN framework and reconstructed the pure-phase object based on a single-shot far-field diffraction pattern.^28^ Typically, such networks use two-dimensional (2D) spatial images as training objects and extract spatial feature information.

In this study, we develop a spatiotemporal absorption fluctuation imaging (ST-AFI) to achieve full-field optical angiography based on U-Net, which utilizes not only the spatial information of the absorption difference between red blood cells (RBCs) and the background, but also the temporal dynamic characteristics resulting from RBC motion. Our method exhibits two key advantages: First, U-Net is used to determine the dynamic imaging of blood flow. Second, spatial and temporal information is analyzed simultaneously using U-Net; thus, this method is not simply a combination of traditional spatial and temporal analyses.^29^ The proposed approach enriches the detected features and ensures high spatial and temporal resolutions for ST-AFI.

## 2 Materials and Methods

### 2.1 Data Acquisition

We used the same experimental imaging setup as that in a previous study.^30^ Low-coherence light with a central wavelength of 540 nm, bandwidth of 10 nm, and power of 100 mW was used to illuminate the sample. Validatory experiments were conducted using 2.5-day-old chicken embryos, which served as live biological samples. Raw blood flow images with a resolution of 256×256  pixels were captured using high-performance telecentric lenses (magnification: 1.7×, #63-232, Edmund Optics) and a high-speed complementary metal oxide semiconductor camera (acA2000-340 km, Basler, Germany). The exposure time and sampling rate of the camera were set to 100  μs and 500 fps, respectively. The camera had a pixel size of 5.5  μm×5.5  μm, and thus, the imaged area was 0.83  mm×0.83  mm. Six datasets with 1024-frame raw blood flow images were employed for the data preparation to obtain the training data. All components of the imaging setup were fixed on a vibration-isolation optical platform. During the experiments, the biological samples were handled carefully in accordance with the laboratory animal protocol approved by the Institutional Animal Care and Use Committee of Foshan University.

### 2.2 Spatiotemporal Absorption Fluctuation Imaging

**Fig. 1:**
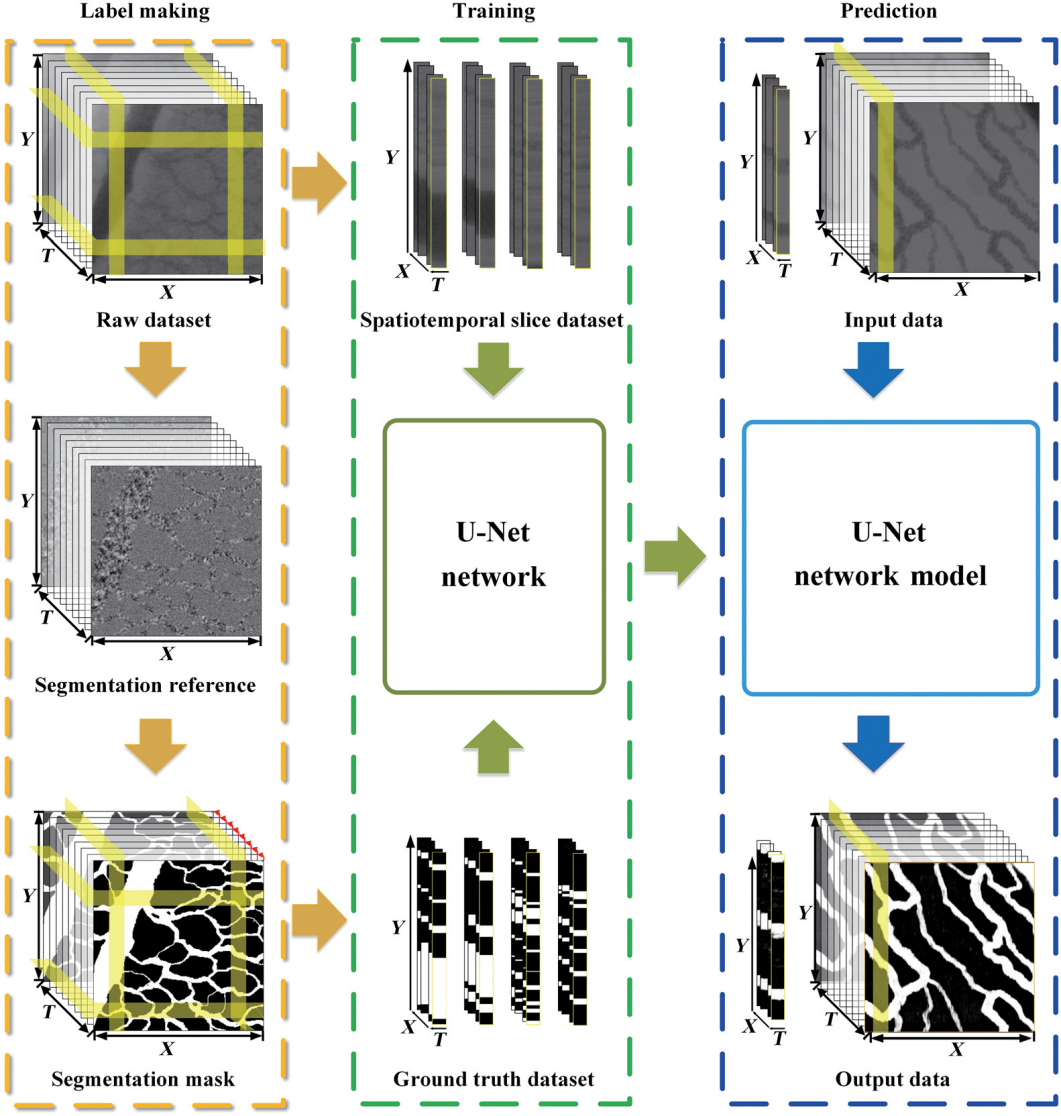
Schematic of the ST-AFI algorithm, including label preparation, U-Net model training, and dataset prediction. The yellow rectangle indicates the acquisition of the reliable GT using the covered labeling method, the green rectangle indicates the U-net model training process with spatiotemporal slice dataset, and the blue rectangle indicates the prediction of the spatiotemporal slice images and vascular reconstruction.

ST-AFI is suitable for the dynamic blood flow imaging of biological samples with high water contents, such as chicken embryos. When a low-coherence light source is used, the speckle of the background tissue fluid can be suppressed, and the absorption coefficient difference between the RBCs and the background tissue fluid is high. Therefore, the blood flow signal of any pixel recorded by a camera can be viewed as a temporal sequence of high-absorption signals corresponding to RBC signals and low-absorption signals corresponding to the signals from “gaps” (background tissue fluid). The acquired raw signal can be expressed as follows:

$$I(x,y,t)=[I_{b}(x,y)+I_{n}(x,y,t)+\overset{m}{\underset{i=1}{\sum }}w_{i}(x,y,t){\mathrm{Gaus}}_{i}(x,y,t)],$$ (1)

where $I_{b}(x,y)$ is the signal from the background tissue fluid, which does not vary with time, and $I_{n}(x,y,t)$ is the signal from the non-blood flow region, which exhibits an absorption similar to that of RBCs. $\overset{m}{\underset{i=1}{\sum }}w_{i}(x,y,t){\mathrm{Gaus}}_{i}(x,y,t)$ represents the blood flow signal, where $w_{i}(x,y,t)$ is the weight assigned to the RBCs concentration and ${\mathrm{Gaus}}_{i}(x,y,t)$ is the pulse of the RBCs cluster. Further, m corresponds to the number of pulses. In the ST-AFI, data with dimensions of X×Y×T were acquired, and a slice dataset (T×Y), including information regarding static spatial absorption difference and dynamic temporal absorption fluctuation, was employed with U-Net. Figure 1 shows a schematic of the ST-AFI algorithm, which includes label preparation, U-Net model training, and dataset prediction; these processes are demarcated by yellow, green, and blue rectangles, respectively. A raw dataset comprising T images with dimensions of X×Y was acquired, and augmented slice and the reliable ground truth (GT) (dimensions of T×Y) were used to train the model. Next, using the trained network model, an ST-AFI image was reconstructed based on the prediction of the spatiotemporal slice images obtained from an entirely different raw dataset.

### 2.3 Network Architecture

**Fig. 2:**
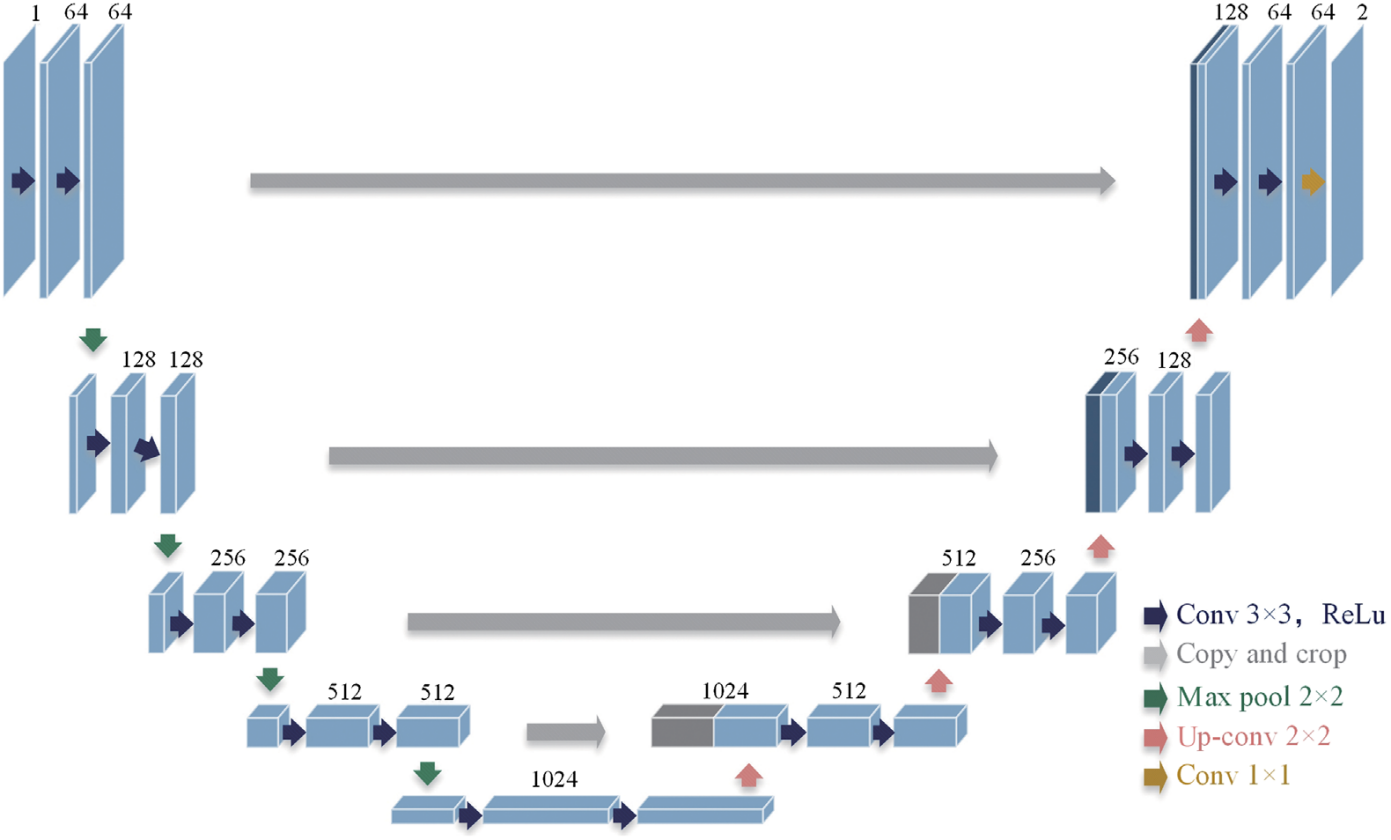
U-Net architecture.

The proposed ST-AFI was performed using the U-Net (Fig. 2).^31^ The network architecture comprises a contractive convolutional encoder (left side) and an expansive convolutional decoder (right side).^32^ The encoder follows the typical architecture of a convolutional network, comprising the repeated application of two 3×3 convolutional layers, each followed by a rectified linear unit (ReLU) activation layer, and a two-stride 2×2 max-pooling layer for downsampling. After each downsampling step, the number of feature channels is doubled, and the image resolution is halved. Every decoder unit performs an upsampling of the feature map; this is followed by a 2×2 convolution (up-convolution) that halves the number of feature channels, a concatenation (skip connections) with the corresponding cropped feature map from the contractive convolutional encoder that preserves the precise localization of extracted data patterns, and two 3×3 convolutions, each followed by a ReLU. At the final layer, a 1×1 convolution is used to map each 64-component feature vector to the desired number of classes. Since a zero padding is applied to each convolutional layer, the width and height of the output image are in accordance with those of the input image.

### 2.4 Training Data Preparation

To ensure that the U-Net would learn the entire spatial and temporal information in the blood flow images, the raw data were preprocessed. As shown in Fig. 1, the raw dataset comprising T consecutive images with dimensions of X×Y was used for label preparation (yellow rectangle), and then, X spatiotemporal slice images with dimensions of T×Y, which were obtained by slicing the images in the X direction, were used for model training (green rectangle).

A labeled image is obtained primarily based on an instantaneous modulation depth (IMD) video. The IMD, which is used to highlight the RBCs motion, is defined as the ratio of the instantaneous signal intensity of the RBCs to that of the background tissue fluid^33^

$$\mathrm{IMD}(x,y,t)=\frac{\mathrm{HPF}[I(x,y,f)]}{\mathrm{LPF}[I(x,y,f)]},$$ (2)

where HPF[ ] and LPF[ ] denote the high-pass filter and the low-pass filter, respectively. The frequency range of the high-pass filter is 1.9 ∼ 190 Hz and that of the low-pass filter is 0 ∼ 1.9 Hz. I(x,y,f) is the frequency domain signal of I(x,y,t). In this study, the IMD video comprised 1024-frame images (256×256).

Owing to the short acquisition time (16 frames, ∼0.03  s), the blood flow distributions of the adjacent acquired raw images are similar. Therefore, the labeled image (X×Y) is copied as the mask along the time sequence to obtain T frames of the consecutive labeled images with a size of X×Y. Subsequently, X frames of the reliable GT with a size of T×Y were input to the U-Net network. The process of labeling GTs by copying labeled images along the time direction and reorganizing the display dimensions of the labeled images is called covered labeling. This labeling method not only improves the label production efficiency but also transforms similar spatial training images into various X-frame training data with spatiotemporal characteristics, which increases the amount of suitable data available for training.

Augmentation assists in training the network effectively and improves the robustness of the network.^34^ As shown in Fig. 1, data augmentation was performed via data rotation (counterclockwise rotations, each of 90 deg). For a particular set of raw data, X slice images, each measuring T×Y, were obtained and then enhanced to a size of 4×T after data augmentation. In this manner, the sparsity of training data was overcome, and the demand for training data was reduced. Moreover, the application of data rotation for model training was found to reduce the impact of uneven light intensity.

### 2.5 Model Training and Angiography

As shown in Fig. 1 (green rectangle), the input dataset for the U-Net model training comprised spatiotemporal slice images (T×Y) obtained from the raw data and the corresponding GTs. Network training was conducted using 6144 augmented slice images with dimensions of 16×256, which were obtained from six sets of raw data. Spatiotemporal slice images from the other four sets of raw data were used for the blood vessel prediction. The training process lasted 50 epochs; 10% of the training data were selected at random for periodic training. In addition, binary cross-entropy was used as the loss function, and the stochastic gradient descent with momentum optimization algorithm was employed. We ran the U-Net model on a PC with an Intel i7-7700 CPU, NVidia GeForce GTX 1660Ti GPU, and 16 GB RAM.

To obtain the ST-AFI images, the trained model was used to predict spatiotemporal slice images not used for training. As shown in Fig. 1 (blue rectangle), X-frame spatiotemporal slice images from a new set of raw data were input to the trained U-Net model to obtain X-frame prediction probability maps (T×Y) of the blood flows; $P_{\mathrm{RBC}}(x,y,t)$ is the prediction probability. The reconstructed ST-AFI image can be expressed as

$$P(x,y)=\mathrm{avr}{\left[P_{\mathrm{RBC}}(x,y,t)\right]}_{t\in [i,j]},$$ (3)

where $\mathrm{avr}{\left[\;\right]}_{t\in [i.j]}$ represents the averaging of $P_{\mathrm{RBC}}(x,y)$ in the region t∈[i,j].

## 3 Results and Discussion

**Fig. 3:**
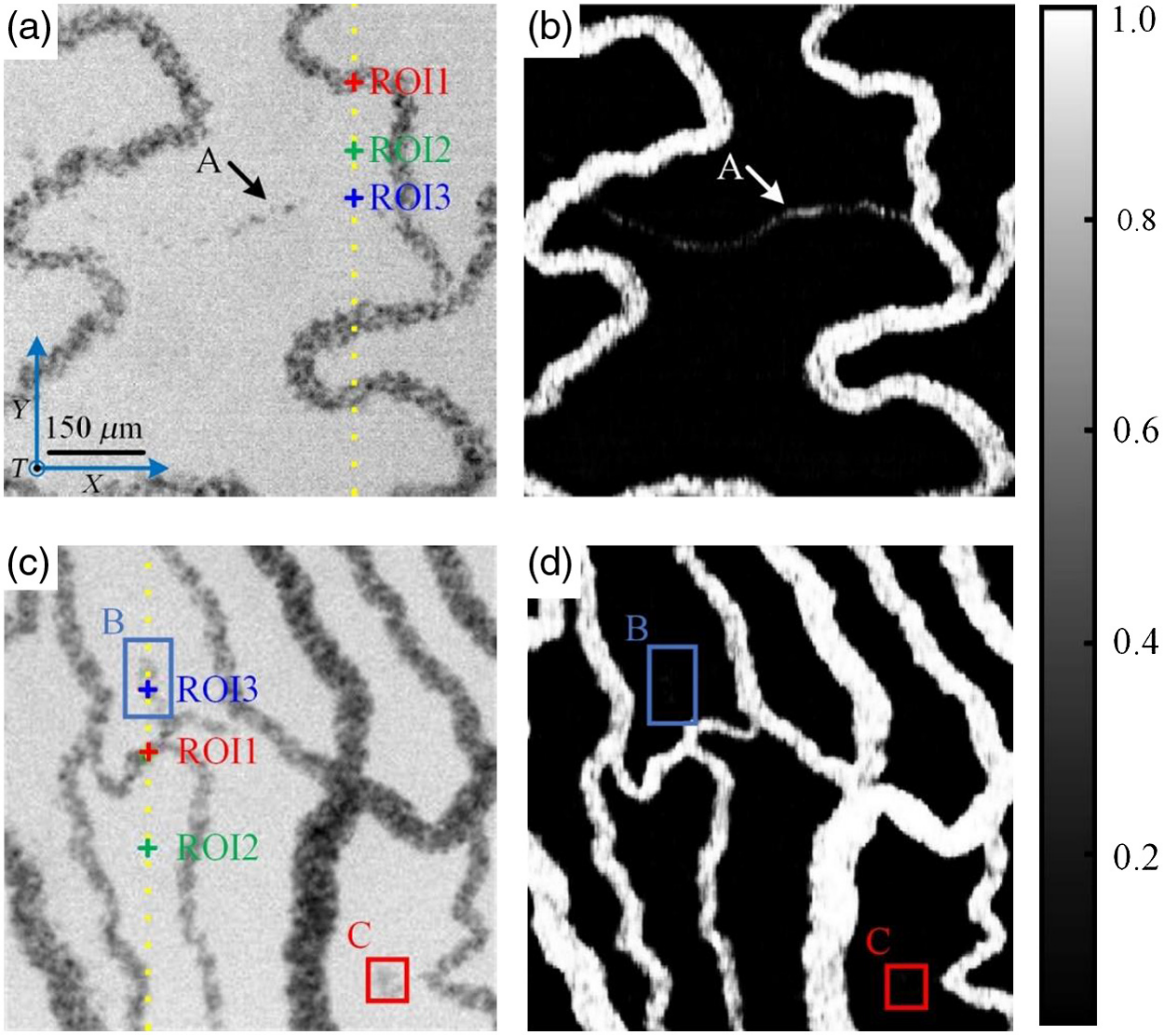
Angiograms obtained using ST-AFI. (a) Raw image 1 (see Video 1). (b) ST-AFI image 1. (c) Raw image 2 (see Video 2). (d) ST-AFI image 2. “A” indicates the capillary with few RBCs flowing intermittently, “B” and “C” indicate static regions with high-absorption coefficients. ROI1–ROI3 in (a) and (c) indicate the regions selected for further analysis. Raw video corresponding to (a), where the dynamic tendency of RBC motion can be observed throughout the field of view (Video 1, AVI, 4.89 MB [URL: https://doi.org/10.1117/1.JBO.27.2.026002.1]). Raw video corresponding to (c), where the dynamic tendency of RBC motion can be observed throughout the field of view (Video 2, AVI, 4.20 MB [URL: https://doi.org/10.1117/1.JBO.27.2.026002.2]).

To demonstrate the physical mechanism of the proposed approach and validate its feasibility, an experiment was conducted using 2.5-day-old chicken embryos, which served as live biological samples. The U-Net model was trained with 6144 spatiotemporal slice images. The two representative results obtained using the model are shown in Fig. 3. The first raw image and reconstructed ST-AFI image of the blood flow in the embryos are shown in Figs. 3(a) and 3(b), respectively. The raw video is shown in Video 1, where the dynamic tendency of RBC motion can be observed throughout the field of view. The video provides clear evidence that the intermittent flow of a few RBCs occurs in the capillaries, as indicated by the arrow “A” in Fig. 3(a). The capillaries were reconstructed continuously, as shown in Fig. 3(b). This was possible because the U-Net extracted not only abundant spatial information but also temporal information. Figure 3(c) shows another raw image. The corresponding ST-AFI image is shown in Fig. 3(d), with rectangles “B” and “C,” recognized as the background; the regions delineated by these rectangles have the same absorption characteristics as blood vessels. Notably, rectangles “B” and “C” in Video 2 do not exhibit any blood flow; these areas could represent background noise or vascular occlusions where microvascular blood flow has completely stopped.

**Fig. 4:**
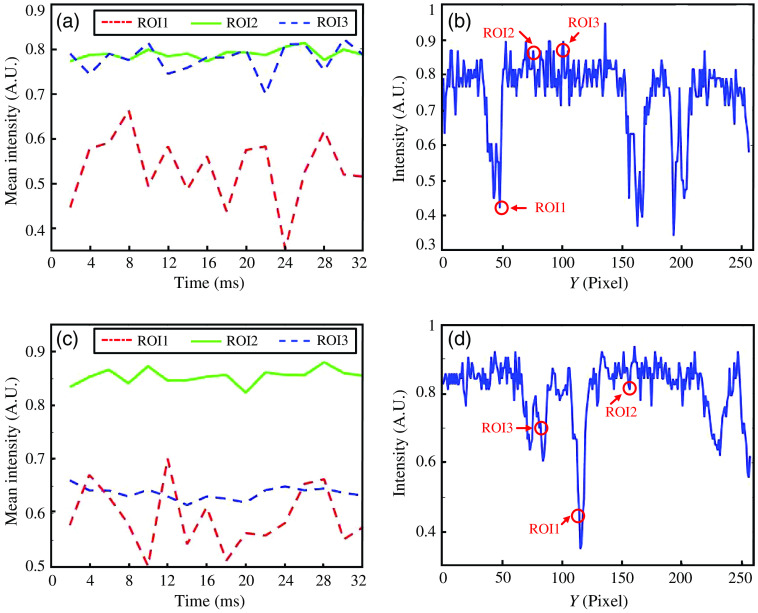
Analysis of the angiograms obtained using ST-AFI. (a) and (c) Mean intensity curves along the ROI1-ROI3 in Figs. 3(a) and 3(c). (b) and (d) Intensity curves along the yellow lines in Figs. 3(a) and 3(c).

The proposed technique can extract capillaries with micro blood flow and suppress non-blood flow regions. This ability is attributed to the spatiotemporal absorption fluctuation effect. The spatiotemporal data of the two images were analyzed, and the results are shown in Fig. 4. The temporal signal curves of the regions of interest 1–3 (ROI1–ROI3), and the spatial signal curve of the yellow line in Fig. 3(a) is shown in Figs. 4(a) and 4(b), respectively. The absorption coefficient of RBCs is considerably higher than that of the background. It is easy to distinguish blood flow and background according to spatial characteristics in Fig. 4(b). However, for capillaries with a low RBCs flow over a short time, the absorption difference between the background and the RBCs can be neglected. Therefore, as shown in Fig. 4(b), the capillaries cannot be easily identified. In such cases, the capillaries can be resolved based on their temporal characteristics. The dynamic absorption signal of moving RBCs [ROI1 and ROI3 in Fig. 4(a)] fluctuates sharply along the time sequence. However, the absorption signal of background tissue fluid [ROI2 in Fig. 4(a)] does not. When spatiotemporal slice images are used to train the network, the features between the dynamic and stationary absorption signals can be recognized. The temporal signal curves of ROI1–ROI3 and the spatial signal curve of the yellow line in Fig. 3(c) are shown in Figs. 4(c) and 4(d), respectively. As in the previous case, blood flow signal [ROI1 in Fig. 4(c)] and background signal [ROI2 in Fig. 4(c)] can be identified based on the spatial characteristics. In addition, an interesting phenomenon occurs. The curve of the non-blood flow region [ROI3 in Fig. 4(d)] shows the pulse of blood flow, but it can be suppressed in the ST-AFI image. When spatiotemporal slice images are input to the U-Net, the temporal characteristics are also extracted simultaneously. The difference between the absorption fluctuation of the RBCs and non-blood flow region helps distinguish between capillaries and non-blood flow regions.

**Fig. 5:**
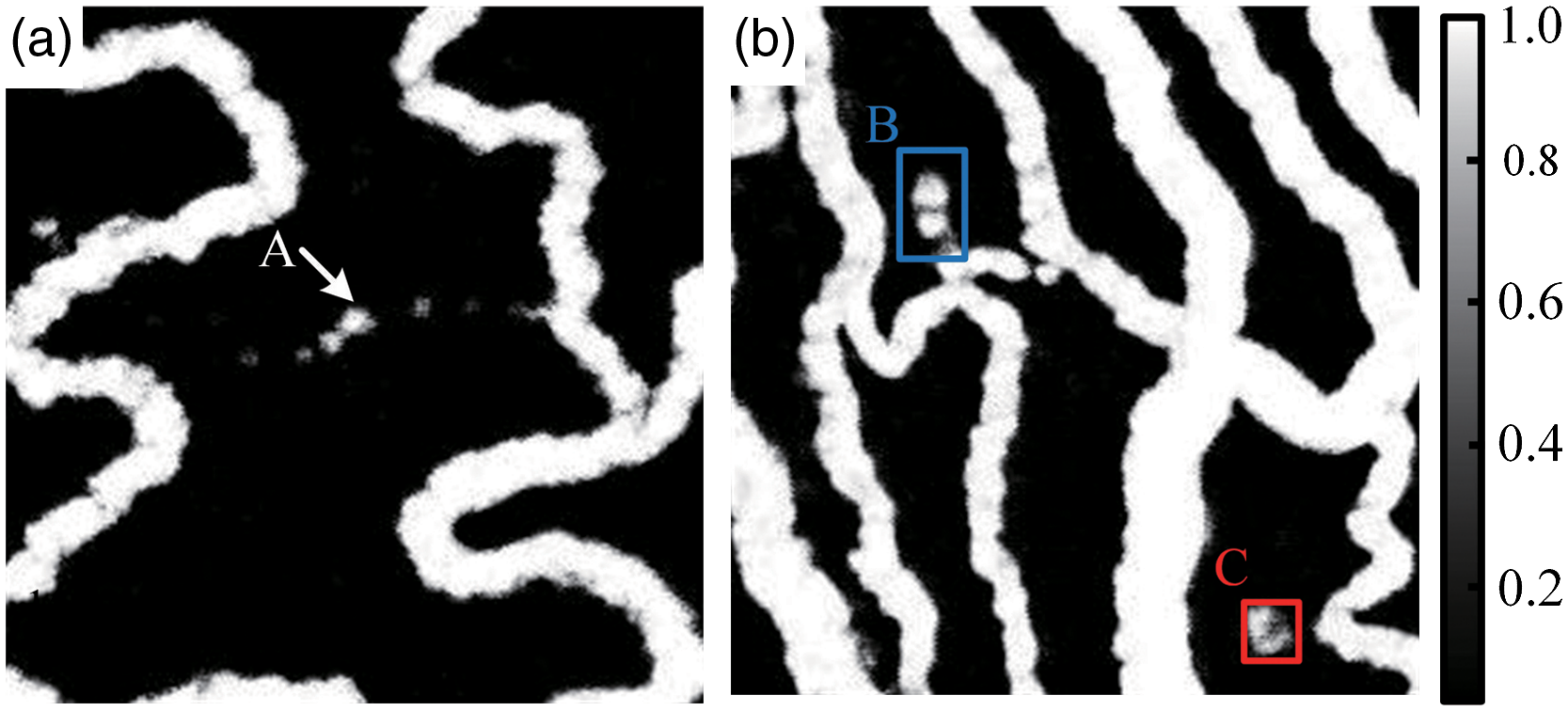
Angiograms obtained using S-AFI. (a) S-AFI image 1 corresponding to raw image 1 [Fig. 3(a)]. (b) S-AFI image 2 corresponding to raw image 2 [Fig. 3(c)].

To highlight the advantages of ST-AFI with spatiotemporal information, a comparative experiment was performed using spatial absorption fluctuation imaging (S-AFI). In S-AFI, the raw spatial blood flow images are used to train the U-Net, and the input data are same as those in ST-AFI. The S-AFI images of the data in Figs. 3(a) and 3(c) are shown in Figs. 5(a) and 5(b), respectively. The small vessel indicated by arrow “A” in Fig. 5(a) is unclear and discontinuous, while the areas indicated by the two rectangles in Fig. 5(b) are considered to depict blood vessels. This is because spatial images do not contain any temporal dynamic absorption fluctuation and because S-AFI cannot resolve continuous capillaries and non-blood flow regions based only on spatial absorption differences.

The accuracy and superiority of the proposed method were validated using quantitative metrics. We calculated four quantitative metrics [accuracy (Acc), sensitivity (Sen), dice coefficient (DC), and intersection over union (IOU)] to evaluate the agreement between the results of ST-AFI and S-AFI and those of the manual delineation of vessels (Table 1). The evaluation metrics are defined as follows:

$$\mathrm{Acc}=\frac{\mathrm{TP}+\mathrm{TN}}{\mathrm{TP}+\mathrm{FP}+\mathrm{TN}+\mathrm{FN}},\mathrm{Sen}=\frac{\mathrm{TP}}{\mathrm{TP}+\mathrm{FN}},\;\mathrm{DC}=\frac{2\times \mathrm{TP}}{2\times \mathrm{TP}+\mathrm{FP}+\mathrm{FN}},\mathrm{IOU}=\frac{\mathrm{GT}\cap \mathrm{SR}}{\mathrm{GT}\cup \mathrm{SR}},$$ (4)

where TP (TN) denotes the number of true positives (true negatives); that is, the number of correctly predicted vessel (background) pixels. Further, FP (FN) denotes the number of false positives (false negatives); that is, the background area (vessel) pixels segmented as vessel (background area) pixels. In addition, GT and segmentation result (SR) were defined to represent the manual segmentation standard and the output of our network, respectively. Based on the results, the proposed method exhibits a high DC and IOU, which means that the angiograms obtained by ST-AFI are consistent with the GT. As expected, S-AFI image 2 [Fig. 5(b)] shows that the high sensitivity of S-AFI was affected by the differences in spatial absorption coefficient in the samples. Considering the aforementioned results, capillary-level dynamic angiography can be achieved using the U-Net to extract information related to spatial absorption coefficient differences and the temporal dynamic absorption fluctuation.

**Table 1:** Agreement (in terms of pixels) between ST-AFI and S-AFI and manual delineation of vessels (mean ± standard deviation).

Image	Acc	Sen	DC	IOU
ST-AFI image 1	0.966±0.003	0.909±0.020	0.921±0.007	0.853±0.012
S-AFI image 1	0.951±0.001	0.901±0.005	0.887±0.002	0.797±0.003
ST-AFI image 2	0.956±0.017	0.933±0.026	0.932±0.026	0.874±0.046
S-AFI image 2	0.945±0.005	0.946±0.004	0.919±0.007	0.850±0.012

**Fig 6:**
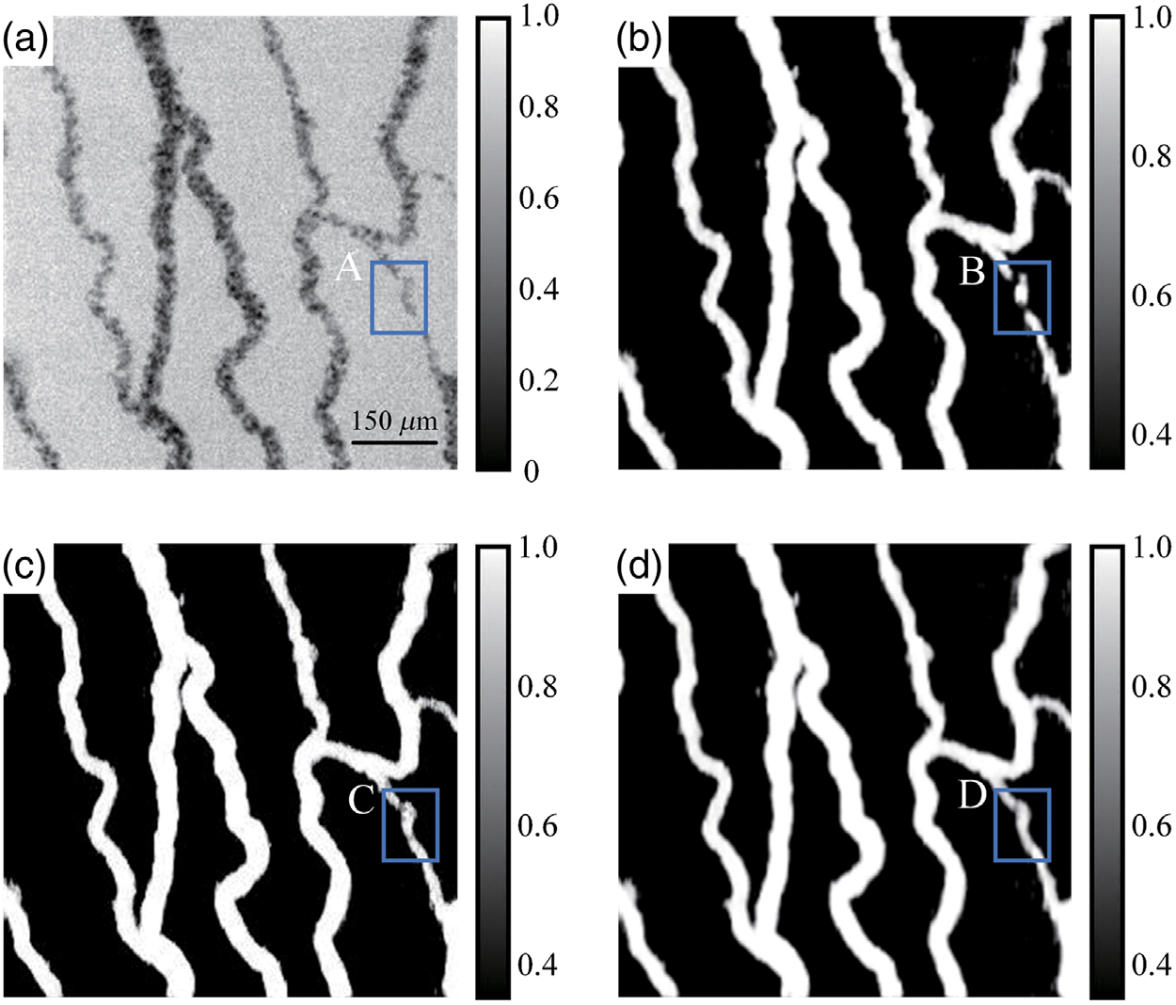
ST-AFI images obtained for T=8, 16, and 32 raw images. (a) Raw image (Video 3). (b)–(d) ST-AFI images obtained for T=8, 16, and 32, respectively. For comparison, “A,” “B,” “C,” and “D” indicate the same ROI. Raw video corresponding to (a), where the dynamic tendency of RBC motion can be observed throughout the field of view (Video 3, AVI, 7.91 MB [URL: https://doi.org/10.1117/1.JBO.27.2.026002.3]).

The amount of raw data in the time direction affects the quality of the imaging process. To select an optimal number of frames, we conducted an experiment using a chicken embryo dataset. Based on Video 3, the position indicated by rectangle “A” in the raw image [Fig. 6(a)] represents vessels with a slow flow velocity and small diameter. To eliminate the influence of the acquisition time on imaging, we acquired 32 raw images, from which we selected 8 and 16 raw images with the same interval. The total acquisition time is 64 ms, which is the enough time taken for an RBC with a diameter of 7  μm to pass through one pixel. Figures 6(b)–6(d) show the results obtained for T=8, 16, and 32 raw images, respectively. Evidently, the proposed method is effective in extracting the temporal and spatial characteristics of blood flow. Additional details are present in the reconstructed blood flow images when several raw images are used. For example, as shown in Figs. 6(b)–6(d), 16 and 32 frames are sufficient to reconstruct vessels with arbitrary blood flow. However, when eight frames are used, only vessels with prominent blood flow characteristics can be resolved, and the vessels indicated by rectangle “A” in Fig. 6(b) cannot be reconstructed successfully. Using more frames assists the U-Net in extracting the temporal dynamic absorption fluctuation characteristics corresponding to microvessels. Therefore, to reduce the difficulty encountered during network training, improve the speed of blood flow image reconstruction, and preserve the main morphological and structural features of blood vessels, we use 16 frames to reconstruct the blood flow images.

**Fig. 7:**
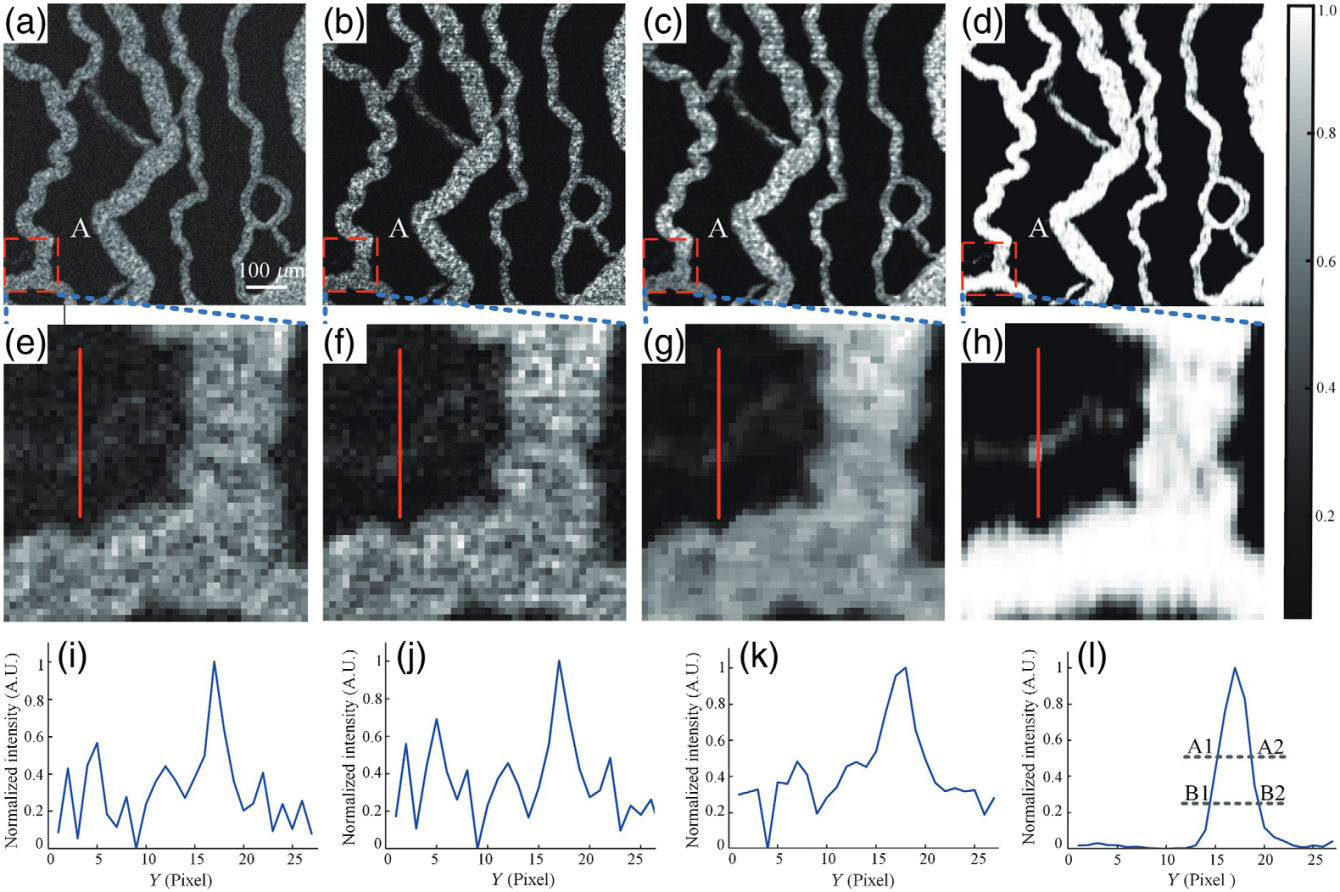
Comparison of angiograms obtained using ST-AFI, TSCA, IFM, and PCA. (a) TSCA image. (b) IFM image. (c) PCA image. (d) ST-AFI image. (e)–(h) Enlarged view of the areas enclosed by the red dashed rectangle in (a)–(d). (i)–(l) Normalized intensity at the positions indicated by the red lines in (e)–(h), and A1–A2 and B1–B2 are the widths at the half and quarter height of the peak, respectively. The area enclosed by the red dashed rectangle represents the ROI. Raw video corresponding to Fig. 7, where the dynamic tendency of RBC motion can be observed throughout the field of view (Video 4, AVI, 6.64 MB [URL: https://doi.org/10.1117/1.JBO.27.2.026002.4]).

Next, we performed an experiment to compare the performance of the proposed method with that of traditional blood flow imaging methods (Fig. 7). The corresponding raw video (Video 4) of the chicken embryos was selected as the reference object. Figures 7(a)–7(d) show the angiographic images obtained via temporal speckle contrast analysis (TSCA),^35^ intensity fluctuation modulation (IFM),^36^ PCA,^10^ and the proposed method, respectively. From Video 4, the position indicated by rectangle “A” in the images [Figs. 7(a)–7(d)] clearly depicts a capillary with indirect blood flow. Although all four methods can reconstruct the blood vessels, the ST-AFI image [Fig. 7(d)] exhibits the more homogeneous contrast. To illustrate the blood vessel distribution in detail, Figs. 7(e)–7(h) present enlarged views of the area indicated by the solid-line rectangles in Figs. 7(a)–7(d), respectively. Evidently, the microvessels reconstructed using ST-AFI are more recognizable. To quantify the differences between the enlarged views of the ROIs, we evaluated them in terms of the signal-to-noise ratio (SNR), which is defined as $\mathrm{SNR}=|20\;\log(\langle Q_{s}\rangle /\langle Q_{n}\rangle )|$. | | represents the absolute value, where $\left\langle Q_{S}\right\rangle$ is the maximum signal intensity of the concerned blood flow and $\left\langle Q_{n}\right\rangle$ is the average signal intensity of the background tissue. The SNRs of the positions indicated by the red lines in Figs. 7(e)–7(h) were 8.25, 8.46, 10.34, and 37.37 dB, respectively. Thus, high-SNR imaging can be realized using the proposed U-Net architecture to extract spatiotemporal signals from blood flow images. Further, we calculated the spatial resolution of the microvessel along the red line in Figs. 7(e)–7(h), which are 11.18, 8.58, 9.25, and 4.33  μm, respectively.^9^ In addition, we quantified the performance of the compared methods with regard to blood flow imaging based on the four previously mentioned evaluation metrics: Acc, Sen, DC, and IOU (Table 2). The proposed method performs well in terms of these metrics. Therefore, the proposed method considerably outperforms TSCA, IFM, and PCA.

**Table 2:** Agreement (in terms of pixels) between the ST-AFI and traditional blood flow imaging methods and manual delineation of blood vessels (mean ± standard deviation).

Method	Acc	Sen	DC	IOU
**TSCA**	0.936	0.837	0.909	0.834
**IFM**	0.953	0.882	0.936	0.879
**PCA**	0.895	0.729	0.843	0.729
**ST-AFI**	**0.962 ± 0.009**	**0.920 ± 0.031**	**0.949 ± 0.013**	**0.903 ± 0.024**

Based on the foregoing analysis of the experimental results, the proposed method provides satisfactory angiograms with high spatial and temporal resolutions. This is achieved by extracting the spatiotemporal characteristics of blood flow images. As each input spatiotemporal slice image used for training reflects the differences in spatial absorption as well as temporal dynamic absorption fluctuation, the trained network model can accurately identify blood vessels and background tissues. In contrast, TSCA acquires images by analyzing the first-order time statistical characteristics of speckle images. This results in a poor temporal resolution and the images are influenced by the concentration and velocity of the moving particles. With IFM, images are obtained by separating the background (static) and blood flow (dynamic) signals. The static signal of the finite time series becomes a *sinc* function after undergoing a Fourier transform; thus, when the time-domain data are limited, the frequency-domain break of the target dynamic signal is included in the background region and cannot be separated. Therefore, the image quality is relatively low. Finally, PCA decomposes the time-domain signal into orthogonal components through an orthogonal transformation to convert raw correlated variables into linearly uncorrelated variables. However, this method can only be used with sensitive imaging techniques, such as laser speckle imaging.

The proposed dynamic blood flow imaging method based on a U-Net achieves a high spatial–temporal resolution as well as a high SNR. However, absorption fluctuation-based imaging is only suitable for transparent samples, such as chicken embryos and zebra fish. For near-turbid tissues, owing to high scattering, raw absorption images are dimmed, and their SNR is reduced. In future research, this limitation will be addressed by increasing the network complexity or substituting the simple CNN architecture with a deep CNN. The proposed approach can be applied to increase the spatiotemporal imaging resolution of other optical imaging methods, such as LSI and optical coherence tomography angiography (OCTA). Our long-term goal is to investigate whether the proposed network can be modified further to develop a multimodal blood flow imaging system that can be integrated with AFI, LSI, and OCTA.

## 4 Conclusion

We proposed a high-quality angiography method based on a U-Net that can reconstruct angiograms from AFI. The 2D spatiotemporal blood flow data were used to train the U-Net model, which analyzed the prediction dataset and yielded reconstructed angiograms. This method uses a neural network to extract information related to the spatial absorption coefficient differences and the temporal dynamic absorption fluctuation of blood flow data. Moreover, it reduces the demand for raw data as well as the extent and difficulty of label production. The experimental results demonstrate that the proposed method not only achieves high-SNR, high-spatial-contrast blood flow imaging, but also suppresses background noise and stagnant vessels by realizing truly dynamic blood flow imaging. As such, this method provides a new avenue for training network models for CNN-based angiography. Consequently, it exhibits considerable potential to be used in studies on biological tissue microcirculation and pathophysiology.

## Code, Data, and Materials Availability

Data to support the findings can be issued on reasonable request to the corresponding author.
